# Survival outcomes and quality of life after percutaneous cryoablation for liver metastasis: A systematic review and meta-analysis

**DOI:** 10.1371/journal.pone.0289975

**Published:** 2023-08-16

**Authors:** Shaghayegh Khanmohammadi, Amir Hossein Behnoush, Shahram Akhlaghpoor

**Affiliations:** 1 Research Center for Immunodeficiencies, Children’s Medical Center, Tehran University of Medical Sciences, Tehran, Iran; 2 Endocrinology and Metabolism Population Sciences Institute, Non-Communicable Diseases Research Center, Tehran University of Medical Sciences, Tehran, Iran; 3 School of Medicine, Tehran University of Medical Sciences, Tehran, Iran; 4 Department of Radiology, Pardis Noor Medical Imaging Center, Tehran, Iran; Humanitas Clinical and Research Center - IRRCS, ITALY

## Abstract

**Background:**

Liver metastasis is present in a wide range of malignancies, with colorectal cancer as the most common site. Several minimally invasive treatments have been suggested for managing hepatic metastases, and cryoablation is among them, yet not widely used. In this systematic review, we aimed to assess the effectiveness of percutaneous cryoablation in all types of liver metastases.

**Methods:**

A systematic search was performed in international databases, including PubMed, Scopus, Embase, and Web of Science, to find relevant studies reporting outcomes for percutaneous cryoablation in liver metastasis patients. In addition to baseline features such as mean age, gender, metastasis origin, and procedure details, procedure outcomes, including overall survival, local recurrence, quality of life (QoL), and complications, were extracted from the studies. Random-effect meta-analysis was performed to calculate the mean difference (MD) and 95% confidence interval for comparison of QoL.

**Results:**

We screened 2131 articles. Fifteen studies on 692 patients were included. Mean overall survival ranged from 14.5–29 months. The rate of local recurrence in the included studies ranged from 9.4% to 78%, and local control progression-free survival ranged from 1 to 31 months. The total QoL decreased one week after the cryoablation procedure (-3.08 [95% Confidence interval: -4.65, -1.50], p-value <0.01) but increased one month (5.69 [3.99, 7.39], p-value <0.01) and three months (3.75 [2.25, 5.24], p-value <0.01) after the procedure.

**Conclusion:**

Cryoablation is an effective procedure for the treatment of liver metastases, especially in cases that are poor candidates for liver resection. It could significantly improve QoL with favorable local recurrence.

## 1. Introduction

The liver is a common site for metastasis from various malignancies such as colorectal cancer, lung cancer, melanoma, and breast cancer, among which colorectal cancer is the most common primary site [1]. In the United States, about 5.1% of all patients diagnosed with malignancy have synchronous liver metastases at the time of diagnosis [2], while it reaches 50% in patients with colorectal cancer origin [3]. Several clinical modalities have been established for liver metastases treatment, including liver resection, systemic and local chemotherapy, and radiotherapy [4]. While liver resection is still the main curative option for colorectal liver metastases [5], this is not the case for many others, such as breast cancer and esophageal cancer [6,7].

In recent years, interventional oncology has become very popular for managing primary and secondary liver malignancies due to its ability to improve survival, reduce tumor burden, and low complication rate [8]. So, the emerging role of interventional oncology as a treatment alone, as a bridge to transplantation, or in association with other approaches could not be denied [9,10].

Thermal ablation, including radiofrequency ablation (RFA) or microwave ablation (MWA), is the most popular local minimally invasive method with many publications and studies. However, cold ablation is less considered in the liver and is not extensively available. Percutaneous cryoablation is in situ destruction of tumor cells with low temperatures. Mechanistically, cellular dehydration, protein denaturation, and microcirculatory failure in thawing and freezing cycles are the main pathways the cryoablation affects the tumor [11]. The current method of cryoablation is the administration of probes with the use of circulating cooled fluid or gas, such as nitrogen or argon, which then expands into a gas, creating low temperatures, including the Joule-Thomson effect [12]. It was first suggested that cryoablation might only be used in cases of liver metastases from colorectal cancer; however, several other studies have assessed the procedure’s effects in other types of metastases [13–15]. Many of these studies have shown the efficacy of cryoablation in improving survival and quality of life (QoL). To date, there is no systematic review investigating the role of cryoablation in liver metastases from different origins. In the present systematic review, we aimed to investigate the effectiveness of percutaneous cryoablation in treating liver metastases through a systematic search in the literature and finding relevant studies.

## 2. Methods and materials

This review was conducted in compliance with the review protocol registered on PROSPERO, 2023 CRD42023390082. The Preferred Reporting Items for Systematic Reviews and Meta-Analyses (PRISMA) Statement was followed in this study [16]. An ethics statement is not applicable because this study is based exclusively on published literature.

### 2.1. Literature search

We systematically searched the electronic databases PubMed, Web of Science (ISI), SCOPUS, and Embase for original articles from inception to November 15, 2022.

We created our search strategy in PubMed and subsequently searched other databases through the following medical subject headings (MeSH) terms and free keywords: “Cryoablation” and “Liver metastasis”. The search strategy is available in S1 Table. No filters or limitations were applied to the search. We transferred all records to EndNote software 20 and removed the duplicates.

### 2.2. Selection criteria

In the final analysis, we only included the papers that met all the following criteria: (1) Original studies with a prospective or retrospective design; (2) Studies on patients over 18 years and affected by liver metastasis (solitary or multiple); (3) Liver metastasis treated with percutaneous cryoablation; (4) Studies reporting outcomes associated with survival, QoL, and tumor control and destruction; (5) English-language studies.

We excluded case reports, letters, editorials, book chapters, commentary articles, review articles, and conference abstracts. Papers reporting the efficacy of cryoablation in combination with hepatic resection. Besides, studies reporting the effectiveness of cryoablation in both liver metastasis and primary hepatic tumors were also excluded.

Two reviewers (SK and AHB) screened titles and abstracts for relevant studies based on inclusion and exclusion criteria. After the collection of eligible studies, a comprehensive full-text review and data extraction were conducted by two authors independently. The third reviewer resolved conflicts in the title/abstract screening.

### 2.3. Data extraction

Two reviewers (SK and AHB) extracted specific data in a dedicated electronic spreadsheet (Excel 2016; Microsoft). Conflicts were resolved through consensus. For each included study, the following data were extracted when available: Author name, publication year, study type, sample size by sex, mean age (range and standard deviation [SD]), number of lesions, primary tumor, follow-up period, previous treatments, ablation cycles, number of cryoprobes, guidance method, initial success, local recurrence, local tumor progression, disease-free survival, overall survival, local control progression-free survival, change in QoL, and complications.

### 2.4. Quality assessment

Study Quality Assessment Tools [17] developed by NIH were used to assess the risk of bias in the included studies. Two authors (SK and AHB) independently performed the quality assessment. Discordance in ratings was resolved through discussion between the authors. Each criterion was answered with “Yes,” “No,” or “Other” (cannot determine, not applicable, not reported). After determining the answer to each question, each study was scored as good, fair, or poor. The purpose of the quality assessment was to clarify the robustness of the evidence, not to exclude studies.

### 2.5. Statistical analysis

Descriptive statistics were used in Microsoft Excel 2016. We calculated the numbers and percentages for the tables when they were not reported. All the analyses were performed using STATA (version 17.0, Stata Corp), and a cutoff of <0.05 in *p-value* was considered statistically significant. Q and Higgin’s I2 were used to determine the heterogeneity of the studies. The heterogeneity of ≤25%, 26–75%, and >75% was considered low, moderate, and high, respectively [18]. We used the random-effect model for the meta-analysis to calculate the mean difference (MD and 95% confidence interval (CI). Random-effect model was implemented due to differences in baseline characteristics of populations in included studies. In these cases, it is suggested to use random-effect model regardless of heterogeneity [19].

## 3. Results

### 3.1. Study characteristics

**Fig 1:**
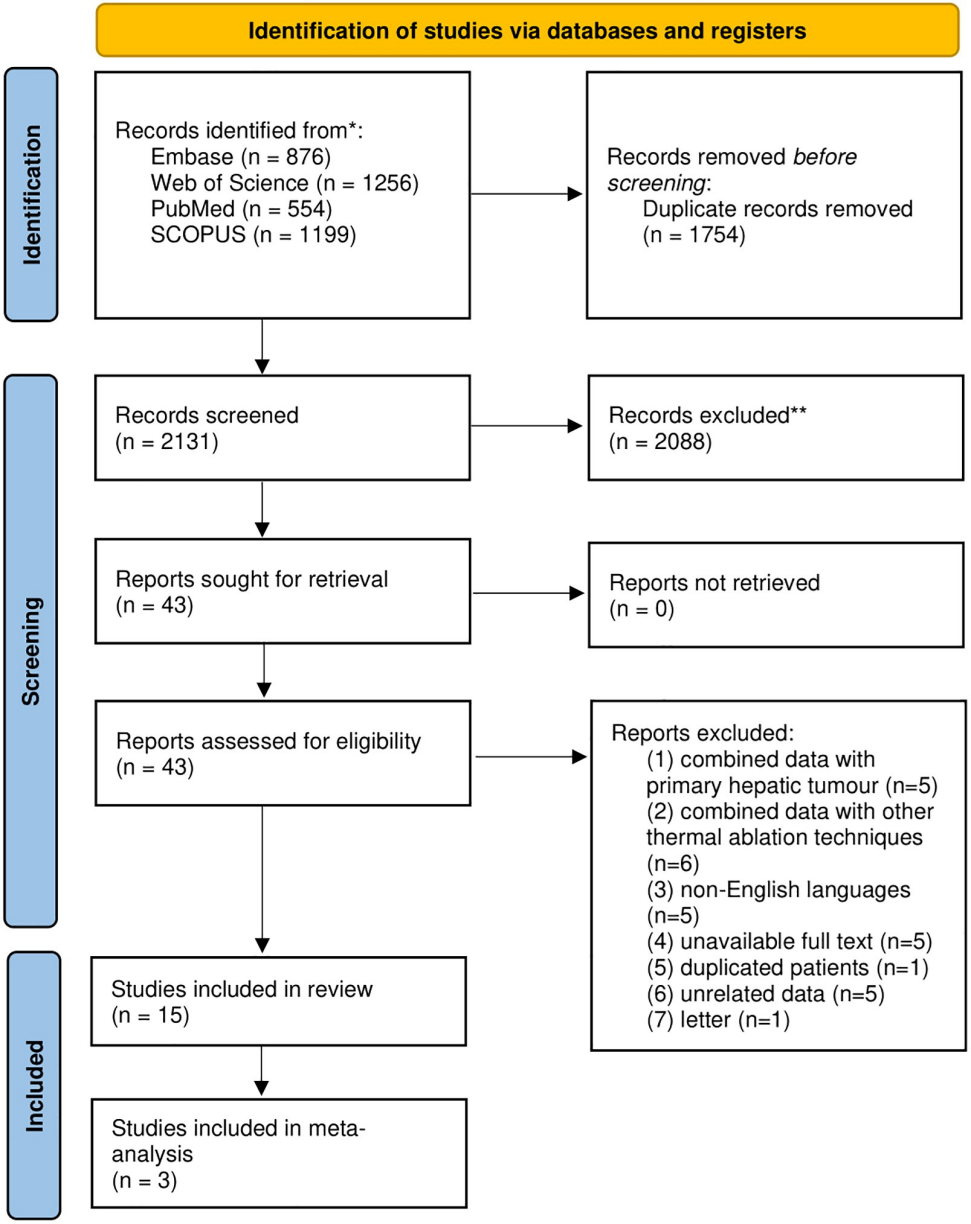
PRISMA flowchart. Flow diagram summarizing the selection of eligible studies based on the PRISMA guidelines.

Our search identified 3,885 publications, including 876 articles from Embase, 1,256 articles from Web of Science, 554 articles from PubMed, and 1,199 articles from Scopus. After removing duplicates, 2,131 records were screened through title and abstract, and 2,088 articles were removed. We reviewed the full text of 43 articles and excluded 28 articles (S2 Table) due to the following reasons: (1) combined data with primary hepatic tumors (n = 5), (2) combined data with other thermal ablation techniques (n = 6), (3) non-English languages (n = 5), (4) unavailable full text (n = 5), (5) duplicated patients (n = 1), (6) unrelated data (n = 5), (7) letter (n = 1). Finally, 15 articles were included in our study [13–15,20–31]. Fig 1 illustrates the flow diagram of study selection.

The baseline characteristics of the included reports are demonstrated in Table 1. Overall, nine retrospective and six prospective studies were included. The total number of patients was 692 (the smallest sample size was 6, and the largest was 326), and 804 lesions were treated. One study did not determine the exact number of patients with liver metastasis [24], and three studies did not report the exact number of lesions [22,28,31]. The patients’ mean age in the studies ranged from 29 to 88. The follow-up period ranged from 0 months to 14.6 years; Hepatic resection, chemotherapy, hormonal therapy, and targeted therapy were among the previous treatments of the included patients. Colorectal cancers were the most common primary tumor site in the included studies. Computed tomography (CT) scan, ultrasound (US), and magnetic resonance imaging (MRI) were used as guidance methods, and the number of cryoprobes ranged from 1 to 5.

**Table 1:** Basic characteristics of the included studies.

ID	Author, year	Study type	Sample size (M/F)	Mean age (SD) (range)	No. of lesions	Primary tumor	Follow-up period	Previous treatment	Ablation cycle (min) goal	Mean number of cryoprobe	Guidance method
1	Adam, 2002 [20]	retrospective	15		16	colorectal, neuroendocrine, bile duct, sarcoma	2–36 months	chemotherapy, hepatic resection	f/t/f/t		US
2	Bang, 2012 [21]	prospective	6		9	lung	11 (0–60) months	chemo/targeted therapy		3.4	CT, US
3	Chang, 2018 [15]	retrospective	19 (14/5)	58.79 (10.79) (38–77)	27	stomach	50 months	chemotherapy	f/t/f/t: 15/5/15/5		CT
4	Feifel, 1998 [22]	prospective	13	61 (31–73)		colorectal, ovary	12.7 (3.32) months	chemotherapy, resection		2–5	US
5	Gao, 2015 [23]	retrospective	13 (0/13)	54 (32–68)	28	ovary	15 (5–22) months	chemotherapy, cytoreduction	f/t/f/t		CT
6	Glazer, 2017 [24]	retrospective		29–88	209	colorectal (61), ovary (29), GIST (21), breast (14), RCC (11), lung (8), esophageal (7), neuroendocrine (9)	2.5 (2 months to 14.6 years) years	hepatic resection	f/t/f 15/10/15		CT, MRI, PET/CT
7	Li, 2014 [31]	retrospective	32 (22/10)	55.2 (29–77)		colorectal			f/t/f/t		CT, US
8	Littrup, 2016 [25]	prospective	176		370	colorectal (178), sarcoma (49), carcinoid (27), pancreas (16), ovary (15), lung (16), renal (13), breast (13), uterine (8), esophagus (6), vagina (5), head and neck (4), bladder (2), prostate (2), multiple myeloma (1), melanoma (1), SCC (1), thyroid (1), cervix (1)	1.8 years			4.5	CT
9	Mala, 2001 [26]	prospective	6	69.5 (8.48) (55–81)	12	colorectal	max 11 months	Open/laparospcopic liver resection, open/laparoscopic cryotherapy	f/t/f/t 20/10-16/20/10-15	2.83	MRI
10	Pusceddu, 2022 [30]	retrospective	38 (14/24)	67.4 (10.8)	50	colorectal (23) Breast (12), pancreas (7), lung (3), thyroid (2), gastric (1), ovary (1), cervix (2)	19.8 (1–60) months		f/t/f/t 4/4/4/4		CT
11	Schuder, 1998 [27]	prospective	6 (2/4)	53 (15.23) (30–69)	8		5–27 months			3	US
12	Silverman, 2004 [29]	retrospective	9 (4/5)	50–81	9	colorectal (5), esophagus (1), stomach (1), lung (1), unknown (1)	12.7 (2–27) months				MRI
13	Wang, 2019 [14]	retrospective	16 (16/0)	median: 60 (45–74)	27	esophagus	median: 14.5 months		f/t/f/t 20/10-16/20/10-15	1.75 (median: 2)	CT
14	Xu, 2008 [28]	prospective	326 (243/83)	54.8 (32–84)		colorectal	36 (7–62) months	chemotherapy	f/t/f/t		US or CT
15	Zhang, 2014 [13]	retrospective	17 (0/17)	55 (30–66)	39	breast	median: 15 (4–22) months	chemotherapy, endocrine therapy	f/t/f/t 15/5/15/5	3.2 (2–5)	CT

CT, computed tomography; f/t freezing/thawing; GIST, gastrointestinal stromal tumor; MRI, magnetic resonance imaging; PET, positron emission tomography; RCC, renal cell carcinoma; SCC, squamous cell carcinoma; SD, standard deviation, TACE, transarterial chemoembolization; US, ultrasound.

### 3.2. Local recurrence and tumor progression after cryoablation

The rate of local recurrence in the included studies ranged from 9.4% to 78% (Table 2). This difference could be explained by different follow-up periods among the included studies. Local tumor progression was detected in 13.1% to 21.6% of lesions.

**Table 2:** Treatment outcomes.

ID	Initial success or response	Local recurrence	Local tumor progression	Disease-free survival (months)	Overall survival (months)	Local control progression free survival (months)	Complications
1	complete devascularization after one treatment: 7/11 after > = 1 treatment: 9/11	7 in 9 (nodular or mass-like enhancing components at MRI or CT or focal 18F-FDG avidity at PET/CT located in or contiguous with the ablation zone)					
2		2 in 9 (any recurrence within the ablation zone resulting from an inadequate, sublethal isotherm likely along the tumor rim)			median: 16 (5–50)	median: 8 (3–24)	2 Grade 1/2
3				7.74 (4.65)	18.95 (11.74) (5–50) 1-year: 78.9% 2-year: 43.4% 3-year: 21.7%	6 months: 59.2% 12 months: 23.2%	pain (10), fever (9), increased liver enzyme (6), pleural effusion (2), pneumothorax (1)
4	complete tumor destruction 8/13						
5	lack of enhancement in 1 month: 100%			1 year: 63.6%	1-year: 92.3%		pain (7), fever (5), increased liver enzyme (6), pleural effusion (2), thrombocytopenia (1)
6	lack of enhancement in 3 months: 88.5% (185/209)	23% (48/209) (nodular or mass-like enhancing components at MRI or CT or focal 18F-FDG avidity at PET/CT located in or contiguous with the ablation zone)					
7	Tumor shrinkage (decrease of ≥30% in tumor size) 62.5%				2-year: 71.9%		Increase in liver enzymes
8		CRC: 11.1% (20/177) non-CRC: 9.4% (18/193) local tumor recurrence mean time in CRC: 9.5 months local tumor recurrence mean time in non-CRC: 7.9 months (either ‘‘procedural” within the ice ablation zone, or ‘‘satellite” within 1 cm of the ablation rim to evaluate recurrence patterns)	In 1 year for CRC: 15.2% In 3 years for CRC: 21.6% (local tumor progression on KM curves are based on patients, rather than individually tracked tumors noting local recurrence rates.)				
9	Tumor shrinkage: 3/6	1 in 6		4-, 2-, and 4-months tumor free in three of the patients	1 died after 7 months, and one after 11 months		pleural fluid (4, 1 requiring drainage), biliary leak (1), pain (2 needing opioids)
10	Complete ablation at 1 month: 48 lesions	11/38 (the appearance of new tumor lesions in the remnant liver)	5/38 (increase in the diameter of the treated lesion)				Minor complications (5): pain (2), self-limited liver bleeding (2), freezing sensation (1)
11	complete destruction of tumors ≤4.5cm (5/5 lesion) 95% destruction of >4.5cm tumors (2/3 lesion)	3 of 8 lesions (3 of 6 patients)/hepatic recurrence 2/6					
12	complete response (no evidence of tumor at follow-up): 3/9 partial response (tumors that were not completely ablated but were smaller, stable, or showed growth in only portions at follow-up): 6/9						
13	lack of enhancement: 87.5%	10 with intrahepatic recurrences (7 of them in remnant liver)			14.5 (4–51) 1-year: 56.3% 2-year: 31.3% 3-year: 18.8%	7.5 (1–31)	pain (7), malaise (6), fever (4), increase in liver enzymes (3)
14	Complete response (lesion disappearance or < 25% of original size): 41/280 partial response (> 30% decrease in the sum of the largest diameter of all targeted lesions): 115/280 stable disease (< 30% decrease in the sum of the largest diameter of all targeted lesions): 68/280 progressive disease (an increase of > 20% in the sum of the largest diameter of all targeted lesions): 56/280	41.70% (by histological examination or by combination of size increase of the lesion on ultrasound, CT or PET imaging and increased tumor markers)			29 (range 3–62) 1-year: 78% 2-year: 62% 3-year: 41% 4-year: 34% 5-year: 23%		pain (103), fever (108), increased liver enzymes (124), thrombocytopenia (58), pleural effusion (20)
15			15.4% (6/39) of metastases (nodular or irregular enhancement or new metastases in the liver, observed during follow-up imaging after 1 month)	1-year: 58.3%	1-year: 70.6%		pain (9), fever (8), increased liver enzymes (5), right pleural effusion (3)

CRC, colorectal cancer; CT, computed tomography; KM, Kaplan Meier; MRI, magnetic resonance imaging; PET, positron emission tomography.

### 3.3. Survival outcomes after cryoablation

Local control progression-free survival ranged from 1 to 31 months. One-year disease-free survival rate ranged from 58.3 to 63.6%, and the mean disease-free survival was between 3.67 and 7.74 months. One-, two-, and three-year overall survival rates were 56.3–92.3%, 31.3–71.9%, and 18.8–41% among the studies, and the mean overall survival ranged from 14.5–29 months (Table 2).

Since the type of primary tumor may affect the survival outcome in patients, we compared the result of studies with one type of primary tumor. The highest and lowest one-year overall survival rates were seen in patients with ovarian cancer (92.3) and esophagus cancer (56.3%), respectively. Also, the two-year overall survival rate in patients with colorectal cancer ranged from 62% to 71.9% (Table 2).

### 3.4. Meta-analysis of QoL of patients before vs. after cryoablation

**Fig 2:**
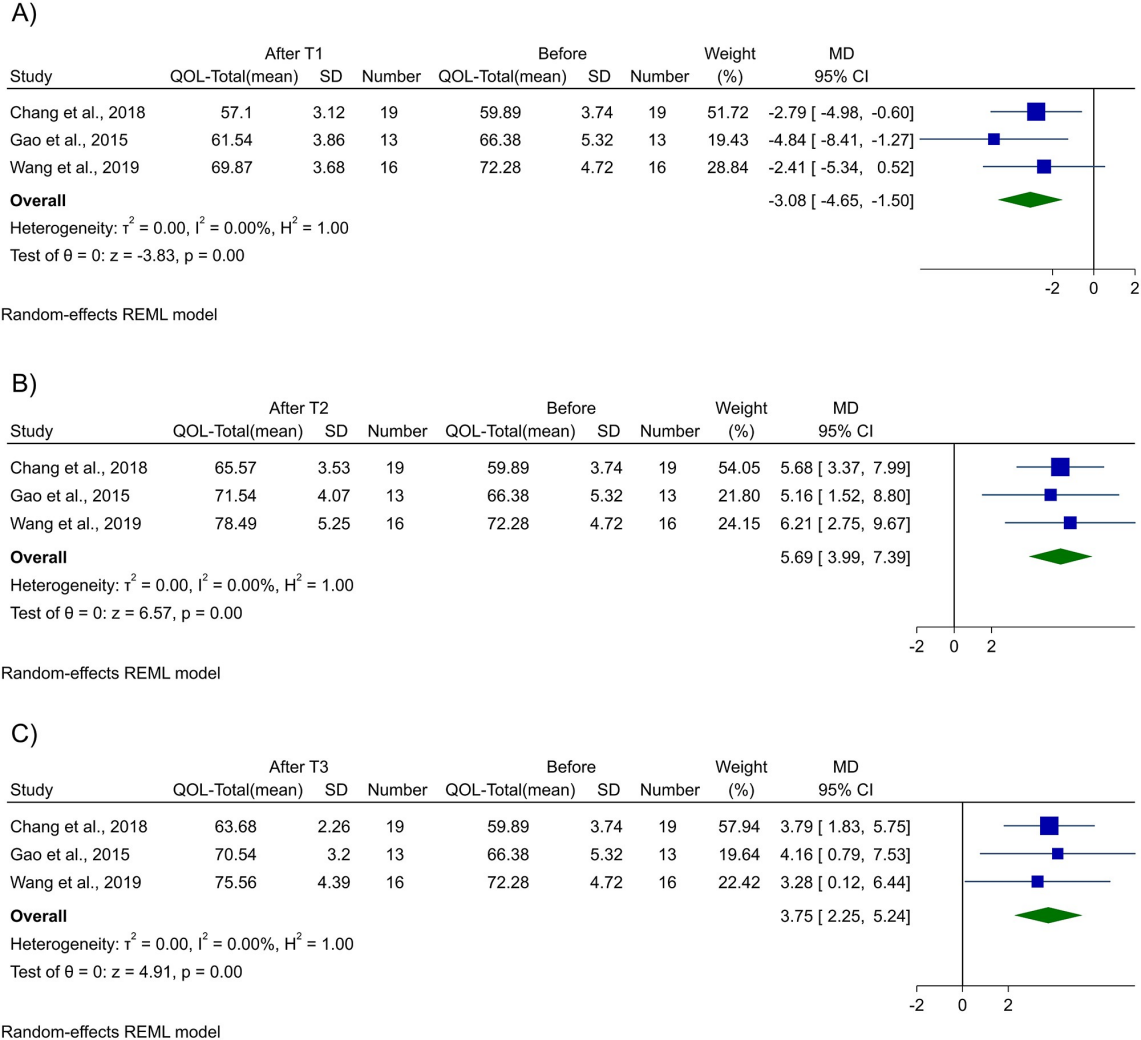
Forest plot. Forest plot for meta-analysis of Quality of Life after A) 1 week, B) 1 month, and C) 3 months of cryoablation.

Five studies [13–15,23,31] investigated the change in QoL of patients with liver metastasis after cryoablation; three studies [13–15] were included in the meta-analysis. QoL was calculated using the Functional Assessment of Cancer Therapy (FACT) version 4.0 (https://www.facit.org/measures/FACT-G) questionnaire. The total QoL decreased one week after the cryoablation procedure (-3.08 [95% Confidence interval: -4.65, -1.50], *p-value* <0.01) but increased one month (5.69 [3.99, 7.39], *p-value* <0.01) and three months (3.75 [2.25, 5.24], *p-value* <0.01) after the procedure (Fig 2). Similar to the change in the total QoL of the patients after cryoablation, physical well-being (PWB), functional well-being (FWB), and emotional well-being (EWB) of the patients had a slight decrease one week after the procedure but increased after one and three months after the procedure. Cryoablation had no statistically significant effect on the social well-being (SWB) of the patients (Table 3, S1–S4 Figs).

**Table 3:** Summary of meta-analysis findings for quality of life change 1 week, 1 month, and 3 months after cryoablation.

Quality of life	Mean Difference (95% CI)	p-value	*I*^*2*^ (%)
**1-week**			
PWB	-1.26 (-1.95, -0.58)	<0.01	0
SWB	-0.03 (-0.98, 0.91)	0.95	35.76
EWB	-0.76 (-1.42, -0.11)	0.02	0
FWB	-1.29 (-1.83, -0.75)	<0.01	0
**Total**	**-3.08 (-4.65, -1.50)**	**<0.01**	**0**
**1-month**			
PWB	2.85 (2.07, 3.63)	<0.01	0
SWB	-0.42 (-2.03, 1.20)	0.61	78.17
EWB	1.43 (0.80, 2.07)	<0.01	0
FWB	2.27 (1.68, 2.87)	<0.01	0
**Total**	**5.69 (3.99, 7.39)**	**<0.01**	**0**
**3-months**			
PWB	2.14 (1.33, 2.95)	<0.01	0
SWB	-0.60 (-1.98, 0.77)	0.39	72.48
EWB	1.04 (0.42, 1.66)	<0.01	0
FWB	1.82 (0.96, 2.67)	<0.01	0
**Total**	**3.75 (2.25, 5.24)**	**<0.01**	**0**

95%CI, 95% confidence interval; EWB, emotional well-being; FWB, functional well-being; PWB, physical well-being; SWB, social well-being.

### 3.5. Complications

Eight studies [13–15,21,23,26,28,30,31] reported complications of cryoablation, exclusively in patients with liver metastasis (i.e., other studies reported complications of cryoablation in both primary tumors and liver metastasis). Considering this fact, reporting the exact rate of complications was not possible in this systematic review. Moreover, most studies did not report the grade of the complications. Increased liver enzymes (144), pain (140), fever (134), thrombocytopenia (59), pleural effusion (31), malaise (6), self-limited liver bleeding (2), grade1/2 complications (2), freezing sensation (1) pneumothorax (1), and biliary leak (1) were among the post-procedure complications (Table 2).

### 3.6. Quality assessment

The result of the study quality assessment is summarized in Table 4. Overall, based on the NIH criteria, ten studies scored as good, two studies as fair, and three studies as poor.

**Table 4:** Quality assessment of included papers.

Questions/ID	#1	#2	#3	#4	#5	#6	#7	#8	#9	#10	#11	#12	#13	#14	#15
Q1	Yes	Yes	Yes	No	Yes	Yes	Yes	Yes	Yes	Yes	Yes	Yes	Yes	Yes	Yes
Q2	Yes	Yes	Yes	No	Yes	No	Yes	Yes	Yes	Yes	Yes	Yes	Yes	Yes	Yes
Q3	Yes	Yes	Yes	CD	Yes	Yes	Yes	Yes	Yes	Yes	Yes	Yes	Yes	Yes	Yes
Q4	Yes	Yes	Yes	Yes	Yes	Yes	Yes	Yes	Yes	Yes	Yes	Yes	Yes	Yes	Yes
Q5	No	No	No	No	No	No	No	Yes	No	No	No	No	No	Yes	No
Q6	Yes	Yes	Yes	Yes	Yes	Yes	Yes	Yes	Yes	Yes	Yes	Yes	Yes	Yes	Yes
Q7	Yes	Yes	Yes	Yes	Yes	Yes	yes	Yes	Yes	Yes	Yes	Yes	Yes	Yes	Yes
Q8	Yes	NR	NR	NR	NR	Yes	NR	NR	NR	NR	NR	NR	NR	NR	Yes
Q9	Yes	Yes	Yes	Yes	Yes	Yes	Yes	Yes	Yes	Yes	Yes	Yes	Yes	Yes	Yes
Q10	Yes	No	Yes	No	Yes	Yes	Yes	Yes	No	No	No	Yes	Yes	Yes	Yes
Q11	Yes	Yes	Yes	Yes	Yes	Yes	Yes	Yes	No	Yes	No	Yes	Yes	Yes	Yes
Q12	CD	CD	CD	CD	CD	NR	CD	CD	CD	CD	CD	CD	CD	CD	NR
Total	Good	Fair	Good	Poor	Good	Good	Good	Good	Poor	Good	Poor	Fair	Good	Good	Good

CD, cannot determine; NR, not reported.

## 4. Discussion

The present systematic review and meta-analysis assessed the effectiveness of percutaneous cryoablation on liver metastasis. With the inclusion of 15 studies ranging from 1998 to 2022 and mostly from colorectal origin, we demonstrated that despite some mainly minor complications, such as the increase in liver enzymes and pain, cryoablation could be a suitable option in liver metastasis cases, mostly due to increase in QoL. Cryoablation reduced local recurrence to less than half in most studies, and local tumor progression was observed in less than one-fourth of the patients. Although the survival of patients ranged from 14.5 to 29 months in different studies, percutaneous cryoablation enhanced the QoL of patients, especially in the first three months.

For many years, liver resection was considered as the most effective treatment for many liver metastases, especially from colorectal origins [32]. This is while only 10–30% of these patients are eligible for liver resection, mainly due to general health status, disease extent, and anatomical locations [33,34]. In recent years, percutaneous ablation has gained attention for treating liver metastases with a limited tumor burden that might need multiple other interventions. This entity includes RFA, MWA, percutaneous ethanol injection (PEA), laser ablation (LA), and cryoablation. RFA is now widely used in the local control of hepatic malignancies, and its beneficiary effects over liver resection have been demonstrated for HCC; hence, it seems a rational option for the treatment of liver metastases [35–37]. On the other hand, a systematic review demonstrated the same efficacy for hepatic malignancies between cryoablation and RFA [38]. Notably, sharp Ice ball marginal zone (< = 5 mm) makes cryoablation a preferable method in central tumors with adjacent critical structures such as bile ducts.

Cryoablation, as one of these methods, is indicated in cases of unresectable secondary liver lesions, comorbid medical conditions preventing the surgeon from resection, or recurrent metastases [39]. It has the advantage of preserving intracellular contents of damaged tumor cells that can be recognized by the immune system and result in an immune response [40,41]. Hence, a combination of immunotherapy and cryoablation may have a synergistic effect, increasing the treatment’s overall efficacy [12]. In line, two clinical studies had demonstrated favorable results for overall survival for HCC when cryoablation was added to the immunotherapy of allogeneic natural killer (NK) cell infusion and dendritic cell cytokine-induced killer (DC-CIK) cells [42,43]. The term “abscopal effect” was also given to the regression of tumors outside of the irradiated field, which might be increased in combination with immunotherapy and radiation [44]. All of these suggest that our results for the use of cryoablation in metastases to the liver can also improve with the addition of immune checkpoint inhibitors.

The overall survival was reported as high as a mean of 29 months in the study by Xu et al., which assessed 326 patients with colorectal liver metastasis [28], while the lowest survival was reported as 14.5 months in esophagus metastasis cases [14]. Analysis of survival has been performed and compared with liver resection in many studies. Colorectal cancer metastases to the liver have been managed in several ways. Thermal ablation in colorectal liver metastases was first accepted in operable instances and in presence of other comorbidities [45]. A meta-analysis by Hao et al. demonstrated that compared with liver resection, RFA could not lead to better survival despite lower complications [46]. Regarding esophageal cancer metastasis, the studies by Liu et al. [47] reported 50.8% and 21.2% for 1- and 2-year survival, and Andreou et al. [48] showed a 5-year survival of 25% after hepatic resection. Meanwhile, Wang et al. study [14], as one of our included studies, reported 1- and 2-year survival rates of 56.3% and 31.3% for percutaneous cryoablation in 16 cases with esophageal carcinoma. For breast cancer liver metastases, there have been several studies that assessed the efficacy of RFA, in which the median overall survival was reported from 10.9 months to 60 months [49–51]. Moreover, MWA showed a mean survival of 32 months [52,53], and LA resulted in survival as high as 50–51 months [54]. Zhang et al. found a 1-year survival of 70.6% in 17 females with 39 liver metastases from breast cancer [13]. Due to various studies conducted for studies with different original cancerous sites, different stages, settings, and techniques should be considered when comparing the results.

Local recurrence rates ranged from 9.4% to 78% in our study with the highest rate in the study by Adam et al. [20] with metastases from the colorectal, neuroendocrine, bile duct, and sarcoma origin. However, it was as low as 17% in the study by Mala et al. [26] in assessment from colorectal-only origin metastases. Several additional mechanisms have been suggested in the literature that demonstrates the cryoablation-induced release of tumor antigens that can lead to the motivation of tumor-specific immune responses, hence, eliminating distant metastases and reducing recurrences [55].

The assessment of QoL is an inevitable aspect of tumor burden evaluation which can reflect the effects of a particular treatment and patients’ prognosis. Although there is limited data for post-cryoablation QoL in liver metastasis cases, three of our included studies reported QoL based on the FACT questionnaire and one with EORTC QLQ-C30. The former is a reliable and valid questionnaire suggested by Cella et al. [56] that is internationally accepted, and the latter is designed to measure cancer patients’ physical, psychological, and social functions [57]. There was a decrease in QoL after 1-week from cryoablation which could be attributed to the minor complications of ablation, such as post-ablation syndrome, pain, and liver function damage. Hence, the use of liver-protecting agents such as short-acting glucocorticoids may be reasonable for this case. Moreover, for 1-month and 3-month analyses, all components of QoL, except SWB, had increases which are mainly due to a decrease in tumor load and complications resolution. These findings on QoL are of higher importance as it is considered one of the prognosis endpoints, with a high value the same as the survival [58]. The increase in QoL is one of the main underlying reasons for the high effectiveness of cryoablation in liver metastases.

Cryoablation was well tolerated, and there were no fatal complications like hemorrhage, cryo-shock, or liver abscess in most of our studies. Increased liver enzymes, pain, and fever were the most observed complications, which may resolve early after some days of procedure or after supportive treatment. This could be attributed to the careful selection of patients and improvements in the techniques delivered to patients, including precise positioning and anchoring with taking advantage of smaller probes. All of these suggest the safety of the cryoablation procedure as a feasible and minimally invasive modality for liver metastasis patients.

The application of cryoablation has been studied for metastatic diseases. In a multicenter study, Jennings et al. showed that cryoablation is a safe and minimally invasive procedure that can improve QoL and relief pain in bone metastases patients [59]. This should be investigated for other metastases to confirm its efficacy in bone disease. On the other hand, in addition to the potential benefits of cryoablation, the cost of cryoablation may be too high, especially for low- middle-income countries (LMIC). It has been suggested that a single treatment can cost up to $10,000, of which half of them are from single-use parts [60]. Another challenge in LMICs might be the availability of argon gas as one of the main gases used in the cryoablation procedure. Hence, all these should be considered in the evaluation of the possible use of cryoablation.

While being the first systematic review assessing the effectiveness of cryoablation for liver metastases, our study had several limitations which should be mentioned. First, the heterogeneity of included studies in terms of cancer origin, stages, and methods used can influence our results and findings. We were unable to compare the survival of patients based on primary tumor type since most studies did not report the survival outcome for each tumor type. Second, the quality of included studies could not be guaranteed as some had poor qualities; hence, there may be biases in included studies. Third, the small sample sizes of included studies and their observational nature can limit our findings. So, further larger randomized controlled trials are warranted to confirm our findings. Finally, as there were differences between studies in reporting the survival outcomes, we were unable to perform meta-analysis for them and QoL was the only outcome meta-analyzed.

## 5. Conclusion

To conclude, cryoablation seems to be an effective and safe procedure for targeting liver metastasis. It could significantly improve QoL with acceptable local recurrence and survival of 14.5 to 29 months among different studies. Especially in cases that are poor candidates for liver resection, cryoablation should be highly considered.

## Supporting information

S1 Checklist PRISMA 2020 checklist. (DOCX)

S1 Fig Forest plot for the meta-analysis of PWB after A) 1 week, B) 1 month, and C) 3 months from cryoablation. (DOCX)

S2 Fig Forest plot for the meta-analysis of SWB after A) 1 week, B) 1 month, and C) 3 months from cryoablation. (DOCX)

S3 Fig Forest plot for the meta-analysis of EWB after A) 1 week, B) 1 month, and C) 3 months from cryoablation. (DOCX)

S4 Fig Forest plot for the meta-analysis of FWB after A) 1 week, B) 1 month, and C) 3 months from cryoablation. (DOCX)

S1 Table Search details. (DOCX)

S2 Table Excluded papers after full text review. (DOCX)
