# Supplementary material for: Survival outcomes and quality of life after percutaneous cryoablation for liver metastasis: A systematic review and meta-analysis
Source: PLoS One. 2023 Aug 16;18(8):e0289975. doi: 10.1371/journal.pone.0289975 (PMC10431656; doi:10.1371/journal.pone.0289975)
Supplement: S2 Fig — (DOCX) [file pone.0289975.s003.docx]

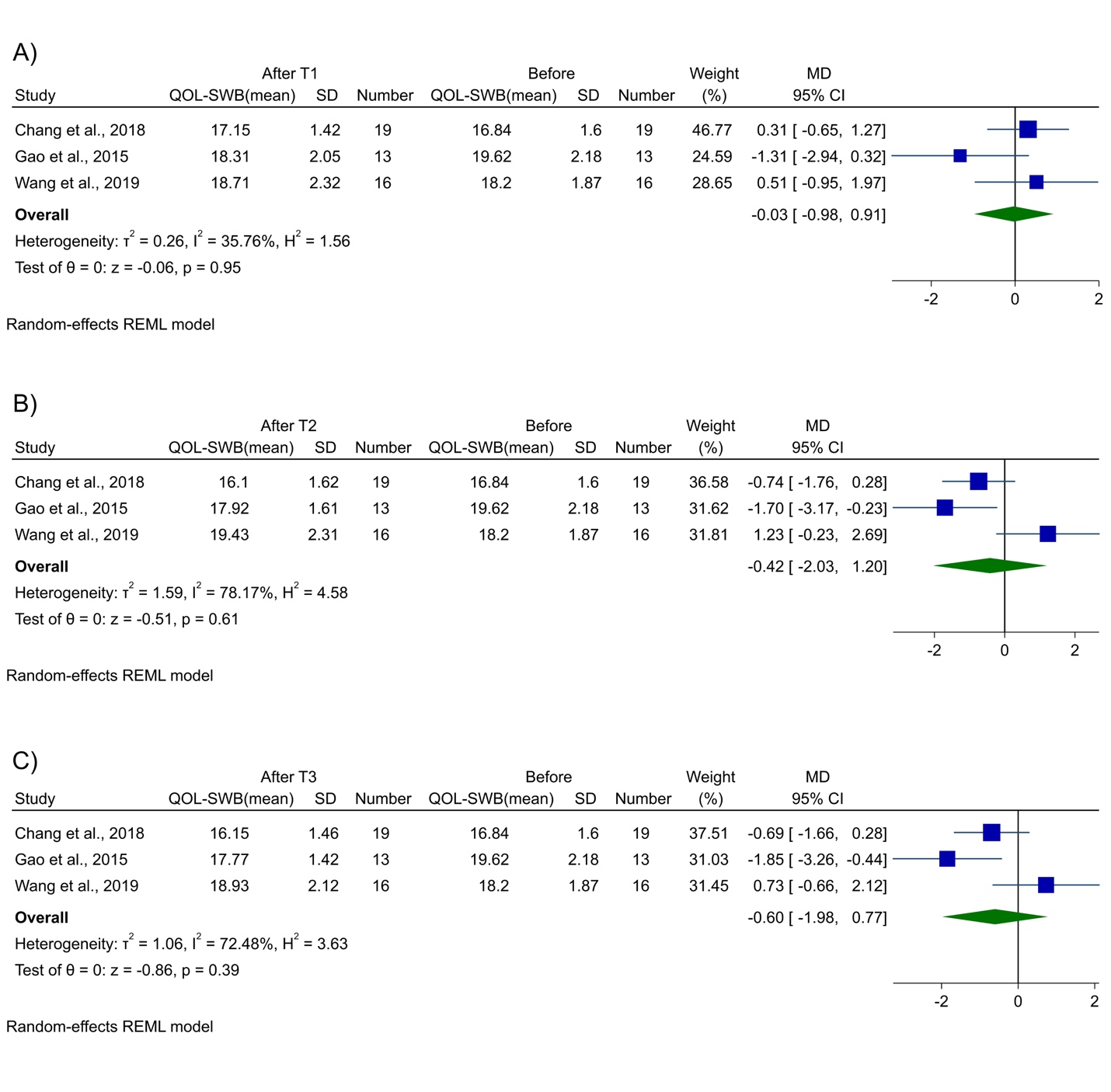


**Supplementary Figure 2.** Forest plot for the meta-analysis of SWB after A) 1 week, B) 1 month, and C) 3 months from cryoablation
