# Supplementary material for: Survival outcomes and quality of life after percutaneous cryoablation for liver metastasis: A systematic review and meta-analysis
Source: PLoS One. 2023 Aug 16;18(8):e0289975. doi: 10.1371/journal.pone.0289975 (PMC10431656; doi:10.1371/journal.pone.0289975)
Supplement: S1 Table — (DOCX) [file pone.0289975.s006.docx]

**Supplementary Materials**

**Supplementary Table 1**. Search details

| Query | | Results  (15 Nov, 2022) |
| --- | --- | --- |
| PubMed | | |
| #1 | (“Cryoablation*”[Title/Abstract] OR “Cryosurgeries”[Title/Abstract] OR “Coablation*”[Title/Abstract] OR “Cryotherapy”[Title/Abstract] OR “Thermal Ablation”[Title/Abstract] OR “Percutaneous Ablation”[Title/Abstract] OR “Image guided Ablation”[Title/Abstract]) | 16,958 |
| #2 | (“Liver metastas*”[Title/Abstract] OR “Hepatic metastas*”[Title/Abstract] OR “secondary liver cancer*”[Title/Abstract]) | 34,388 |
| #3 | #1 AND #2 | 554 |
| SCOPUS | | |
| #1 | TITLE-ABS-KEY(“Cryoablation*” OR “Cryosurgeries” OR “Coablation*” OR “Cryotherapy” OR “Thermal Ablation” OR “Percutaneous Ablation” OR “Image guided Ablation”) | 37,759 |
| #2 | TITLE-ABS-KEY(“Liver metastas*” OR “Hepatic metastas*” OR “secondary liver cancer*”) | 68,778 |
| #3 | #1 AND #2 | 1,199 |
| Embase | | |
| #1 | (“Cryoablation*”:ti,ab,kw OR “Cryosurgeries”:ti,ab,kw OR “Coablation*”:ti,ab,kw OR “Cryotherapy”:ti,ab,kw OR “Thermal Ablation”:ti,ab,kw OR “Percutaneous Ablation”:ti,ab,kw OR “Image guided Ablation”:ti,ab,kw) | 26,655 |
| #2 | (“Liver metastas*”:ti,ab,kw OR “Hepatic metastas*”:ti,ab,kw OR “secondary liver cancer*”:ti,ab,kw) | 52,703 |
| #3 | #1 AND #2 | 876 |
| Web of Science | | |
| #1 | TS=(“Cryoablation*” OR “Cryosurgeries” OR “Coablation*” OR “Cryotherapy” OR “Thermal Ablation” OR “Percutaneous Ablation” OR “Image guided Ablation”) | 21,817 |
| #2 | TS=(“Pain” OR “Painful” OR “Ache*” OR “Management, Pain” OR “Managements, Pain” OR “Pain Management*” “Palliative Care” OR “Analgesia” OR “Palliation”) | 36,848 |
| #4 | #1 AND #2 | 1,256 |

Total: 3885
