# Supplementary material for: Survival outcomes and quality of life after percutaneous cryoablation for liver metastasis: A systematic review and meta-analysis
Source: PLoS One. 2023 Aug 16;18(8):e0289975. doi: 10.1371/journal.pone.0289975 (PMC10431656; doi:10.1371/journal.pone.0289975)
Supplement: S2 Table — (DOCX) [file pone.0289975.s007.docx]

**Supplementary Materials**

**Supplementary Table 2**. Excluded papers after full text review.

| Author, year | Title | Reason of exclusion |
| --- | --- | --- |
| Bang, 2012 [1] | Percutaneous cryoablation of metastatic renal cell carcinoma for local tumor control: Feasibility, outcomes, and estimated cost-effectiveness for palliation | Duplicated patients |
| Barral, 2016 [2] | Percutaneous Thermal Ablation of Breast Cancer Metastases in Oligometastatic Patients | Combined data with other thermal ablation techniques |
| Bilchik, 2000 [3] | Cryosurgical ablation and radiofrequency ablation for unresectable hepatic malignant neoplasms - A proposed algorithm | Combined data with primary hepatic tumors |
| Cao, 2019 [4] | Argon-helium cryoablation for the treatment of hepatic metastases from nasopharyngeal carcinoma: Initial results in 16 patients | Non-English languages |
| Chang, 2018 [5] | Argon-helium knife cryoablation for the treatment of liver metastases from gastric cancer | Non-English languages |
| Daye, 2022 [6] | Periprocedural factors associated with overall patient survival following percutaneous image-guided liver tumor cryoablation | Combined data with primary hepatic tumors |
| Doussot, 2015 [7] | Liver resection and ablation for metastatic melanoma: A single center experience | Combined data with other thermal ablation techniques |
| Guojun 2007 [8] | Percutaneous cryoablation after chemoembolization or radiotherapy for liver malignancy (45 cases report) | Unavailable full text |
| Huang, 2002 [9] | Phase I study of percutaneous cryotherapy for colorectal liver metastasis, | Unrelated data |
| Jungraithmayr, 2005 [10] | Cryoablation of malignant liver tumors: Results of a single center study | Unavailable full text |
| Kalra, 2021 [11] | Percutaneous Cryoablation of Liver Tumors: Initial Experience from a Tertiary Care Center in India | Combined data with primary hepatic tumors |
| Chen, 2019 [12] | Assessment of the cryoablation margin using MRI–CT fusion imaging in hepatic malignancies | Combined data with primary hepatic tumors |
| Long, 2013 [13] | Alleviating the pain of unresectable hepatic tumors by percutaneous cryoablation: Experience in 73 patients | Unrelated data |
| Mala, 2004 [14] | Cryoablation of colorectal liver metastases: minimally invasive tumor control | Combined data with other thermal ablation techniques |
| Matsui, 2003 [15] | Percutaneous cryoablation therapy under local anesthesia for unresectable metastatic liver tumors | Non-English languages |
| Nair, 2008 [16] | Biochemical and hematologic alterations following percutaneous cryoablation of liver tumors: Experience in 48 procedures | Unrelated data |
| Nakazaki, 2001 [17] | Percutaneous cryosurgery for liver tumors | Non-English languages |
| Odisio, 2017 [18] | Planned Treatment of Advanced Metastatic Disease with Completion Ablation After Hepatic Resection | Combined data with other thermal ablation techniques |
| Odisio, 2018 [19] | Impact of Prior Hepatectomy History on Local Tumor Progression after Percutaneous Ablation of Colorectal Liver Metastases | Combined data with other thermal ablation techniques |
| Osada, 2007 [20] | Serum cytokine levels in response to hepatic cryoablation | Unrelated data |
| Pearson, 1999 [21] | Intraoperative radiofrequency ablation or cryoablation for hepatic malignancies | Unrelated data |
| Qian, 2005 [22] | Percutaneous cryoablation in treatment of metastatic liver malignancy: A report of 22 cases | Unavailable full text |
| Ruers, 2007 [23] | Comparison between local ablative therapy and chemotherapy for non-resectable colorectal liver metastases: A prospective study | Combined data with other thermal ablation techniques |
| Seifert, 2002 [24] | Cryotherapy for primary and secondary liver tumours | Non-English languages |
| Shapiro, 2004 [25] | Cryotherapy and percutaneous ablation (multiple letters) | Letter |
| Shyn, 2011 [26] | MRI contrast enhancement of malignant liver tumours following successful cryoablation | Combined data with primary hepatic tumors |
| Xu, 2009 [27] | Percutaneous cryosurgery for the treatment of colorectal liver metastases | Unavailable full text |
| Zhang, 2013 [28] | Cryoablation for salvage therapy of liver metastases: An analysis of 46 cases | Unavailable full text |

1. Bang, H.J., et al., *Percutaneous cryoablation of metastatic renal cell carcinoma for local tumor control: Feasibility, outcomes, and estimated cost-effectiveness for palliation.* Journal of Vascular and Interventional Radiology, 2012. **23**(6): p. 770-777.

2. Barral, M., et al., *Percutaneous Thermal Ablation of Breast Cancer Metastases in Oligometastatic Patients.* Cardiovascular and Interventional Radiology, 2016. **39**(6): p. 885-893.

3. Bilchik, A.J., et al., *Cryosurgical ablation and radiofrequency ablation for unresectable hepatic malignant neoplasms - A proposed algorithm.* Archives of Surgery, 2000. **135**(6): p. 657-662.

4. Cao, F., et al., *Argon-helium cryoablation for the treatment of hepatic metastases from nasopharyngeal carcinoma: Initial results in 16 patients.* Journal of Interventional Radiology (China), 2019. **28**(3): p. 247-251.

5. Chang, X., et al., *Argon-helium knife cryoablation for the treatment of liver metastases from gastric cancer.* Journal of Interventional Radiology (China), 2018. **27**(1): p. 40-44.

6. Daye, D., et al., *Periprocedural factors associated with overall patient survival following percutaneous image-guided liver tumor cryoablation.* Int J Hyperthermia, 2022. **39**(1): p. 34-39.

7. Doussot, A., et al., *Liver resection and ablation for metastatic melanoma: A single center experience.* J Surg Oncol, 2015. **111**(8): p. 962-8.

8. Guojun, Q. and W. Mengchao, *Percutaneous cryoablation after chemoembolization or radiotherapy for liver malignancy (45 cases report).* Technology in Cancer Research and Treatment, 2007. **6**(5): p. 470-474.

9. Huang, A., et al., *Phase I study of percutaneous cryotherapy for colorectal liver metastasis.* Br J Surg, 2002. **89**(3): p. 303-10.

10. Jungraithmayr, W., et al., *Cryoablation of malignant liver tumors: Results of a single center study.* Hepatobiliary and Pancreatic Diseases International, 2005. **4**(4): p. 554-560.

11. Kalra, N., et al., *Percutaneous Cryoablation of Liver Tumors: Initial Experience from a Tertiary Care Center in India.* J Clin Exp Hepatol, 2021. **11**(3): p. 305-311.

12. Chen, C., et al., *Assessment of the cryoablation margin using MRI–CT fusion imaging in hepatic malignancies.* Clinical Radiology, 2019. **74**(8): p. 652.e21-652.e28.

13. Long, X., et al., *Alleviating the pain of unresectable hepatic tumors by percutaneous cryoablation: Experience in 73 patients.* Cryobiology, 2013. **67**(3): p. 369-373.

14. Mala, T., et al., *Cryoablation of colorectal liver metastases: minimally invasive tumour control.* Scand J Gastroenterol, 2004. **39**(6): p. 571-8.

15. Matsui, K., et al., *[Percutaneous cryoablation therapy under local anesthesia for unresectable metastatic liver tumors].* Gan To Kagaku Ryoho, 2003. **30**(11): p. 1591-4.

16. Nair, R.T., et al., *Biochemical and hematologic alterations following percutaneous cryoablation of liver tumors: Experience in 48 procedures.* Radiology, 2008. **248**(1): p. 303-311.

17. Nakazaki, H., et al., *[Percutaneous cryosurgery for liver tumors].* Gan To Kagaku Ryoho, 2001. **28**(11): p. 1599-602.

18. Odisio, B.C., et al., *Planned Treatment of Advanced Metastatic Disease with Completion Ablation After Hepatic Resection.* J Gastrointest Surg, 2017. **21**(4): p. 628-635.

19. Odisio, B.C., et al., *Impact of Prior Hepatectomy History on Local Tumor Progression after Percutaneous Ablation of Colorectal Liver Metastases.* J Vasc Interv Radiol, 2018. **29**(3): p. 395-403.e1.

20. Osada, S., et al., *Serum cytokine levels in response to hepatic cryoablation.* Journal of Surgical Oncology, 2007. **95**(6): p. 491-498.

21. Pearson, A.S., et al., *Intraoperative radiofrequency ablation or cryoablation for hepatic malignancies.* American Journal of Surgery, 1999. **178**(6): p. 592-598.

22. Qian, G., S. Wu, and N. Wang, *Percutaneous cryoablation in treatment of metastatic liver malignancy: A report of 22 cases.* Chinese Journal of Clinical Oncology, 2005. **32**(24): p. 1400-1402.

23. Ruers, T.J.M., et al., *Comparison between local ablative therapy and chemotherapy for non-resectable colorectal liver metastases: A prospective study.* Annals of Surgical Oncology, 2007. **14**(3): p. 1161-1169.

24. Seifert, J.K., A. Heintz, and T. Junginger, *Cryotherapy for primary and secondary liver tumours.* Zentralblatt fur Chirurgie, 2002. **127**(4): p. 275-281.

25. Shapiro, R.S., et al., *Cryotherapy and percutaneous ablation [2] (multiple letters).* American Journal of Roentgenology, 2004. **182**(6): p. 1597.

26. Shyn, P.B., et al., *MRI contrast enhancement of malignant liver tumours following successful cryoablation.* Eur Radiol, 2012. **22**(2): p. 398-403.

27. Xu, K., et al., *Percutaneous cryosurgery for the treatment of colorectal liver metastases.* Chinese Journal of Gastroenterology, 2009. **14**(9): p. 517-521.

28. Zhang, W.H., et al., *Cryoablation for salvage therapy of liver metastases: An analysis of 46 cases.* World Chinese Journal of Digestology, 2013. **21**(11): p. 1024-1028.
